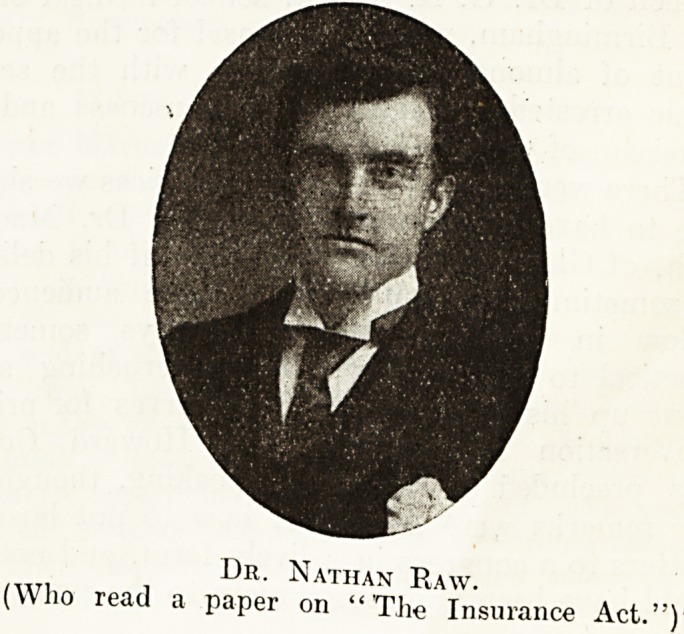# Personalities at the Conference

**Published:** 1912-09-28

**Authors:** 


### PERSONALITIES AT THE CONFERENCE.

### An Impression from the Hearers' Point of View.

As we ventured to predict, immense interest was excited in Birmingham by the discussions and papers which occupied the Conference of the British

Sir William Collins.
(Who read a paper on "Hospitals and the State.")

Hospitals Association last week. Indeed, that interest, as those in the audience could not help anticipating, and as was proved later by the reports which appeared in practically every newspaper throughout the country, was very widespread. For this, no doubt, two causes may be adduced, the tremendous agency for focussing public attention on the work of the voluntary hospitals which the British Hospitals Association has proved to be and, as was very clear to those present, the personalities of those who were responsible for the organisation of the proceedings. Undoubtedly, Mr. Howard Collins, house governor of the General Hospital, Birmingham, and honorary local secretary of the Conference, is the man to whom perhaps more than to anyone thanks should be given for the success of the meetings. Never prominent, his influence pervaded the Council Chamber. The work involved by the local secretaryship of a Conference of this sort is immense and exacting; and as Mr. Collins himself remarked, it was not the want of co-operation but its abundance that was embarrassing. The fine record set originally by Dr. Mackintosh, M.V.O., at the first Conference of the Association in Glasgow three years ago, and ably seconded by Mr. W. G. Carnt at Manchester last year, has been equalled by Mr. Collins, who deserves the thanks of every hospital manager for his conduct of the arrangements. But the best sort of success is never, or hardly ever, a one-man show.

Without the help of the City Corporation even Mr. Howard Collins would have been in difficulties; no one but the Lord Mayor, Alderman Bowater, could have placed the Council House at the Association's disposal, and it is not every Lord Mayor who would have allowed the meetings to be held in the splendid Council Chamber as Alderman Bowater very hospitably did. Nothing could have inaugurated the meetings better than the Lord Mayor's address of welcome, which struck us in the audience as no mere string of polite phrases, but itself a thought-provoking speech, which, as events proved, was somewhat on the lines of Sir William Collins' paper. The keen debate which followed his remarks on "Hospitals and the State" made everyone regret that Sir William himself was unable to reply in person, having to be

Mr. J. B. Clarke.
(President of the Conference.)

The Lord Mayor of Birmingham.

(Ald. Bowater.)

Mr. Howard J. Collins.
(Hon. Local Secretary.)

Dr. Nathan Raw.
(Who read a paper on "The Insurance Act.")

present at two conferences at Geneva, but the paper itself, from the broad aspect with which it dealt with the subject, formed a capital lead off to the subsequent discussions. Still, it quickly became apparent that the good papers would only be followed by good debates if the chairman were a man who knew his business and were capable of piloting the discourse through all the shoals that beset a public discussion. When to interrupt a speaker, when to allow him an excursion of which the general sense of the meeting approves: all this Mr. J. B. Clarke did admirably and very genially. It is a tribute to the practice of hospital chairmanship when we say how popular were Mr. Clarke's presidential rulings, and his experience of the tact required is soon understood when we add that he has been on the committee of the General Hospital, Birmingham, or in the chair, for twenty-one years past.

In taking as his subject "The Probable Effect of the Insurance Act," Dr. Nathan Raw, of Liverpool, had in one sense the most popular subject of the day, if popularity may be gauged by diversity of opinion and general attention. He also represented, very desirably, the views which impress themselves on those who are responsible for the administration of our Poor-Law infirmaries, views of which we think too little has been heard in this controversy hitherto. Dr. Nathan Raw is impressed with the desirability of dealing with sickness on national and municipal lines, and forecasted the provision of special hospitals for insured persons by the Insurance Committees as soon as funds had accumulated sufficiently. His paper was listened to with great attention, and should have a special interest for that great body of readers who are immediately concerned in the problems of Poor-Law infirmary work.

The personal characteristics of the speakers were betrayed in an interesting fashion in the course of the debates, and though to some extent our readers will have what those present had not—namely, a sight of Sir William Collins from the photograph we are privileged to publish. Who could not diagnose the M.P. in the point of view and handling of his paper? Mr. J. B. Clarke again, in his geniality and control of the meetings, proved to everyone that a chairman is made and not born. Again, the capital speech of Dr. G. A. Auden, school medical officer for Birmingham, with its proposal for the appointment of almoners in connection with the school clinic arrested everyone by the terseness and enthusiasm of the speaker.

There were many present whose voices we should like to have heard more frequently; Dr. Mackintosh, of Glasgow, for instance, who if his delivery is sometimes too rapid for a large audience to follow in comfort, yet has always something practical to say, and generally a crushing argument up his sleeve which he reserves for private conversation afterwards. Mr. Howard Collins was precluded from much speaking, though his few remarks were models of how to put business matters to a congress in a lively form, and nothing could have been happier or more to the point than his acknowledgment of the vote of thanks which concluded the proceedings, in which he confessed that this was the one matter which he had not arranged. May the Conference next year, whether held in the provinces or in London, be equally fortunate in its honorary local secretary and personnel!

## Figures and Tables

**Figure f1:**
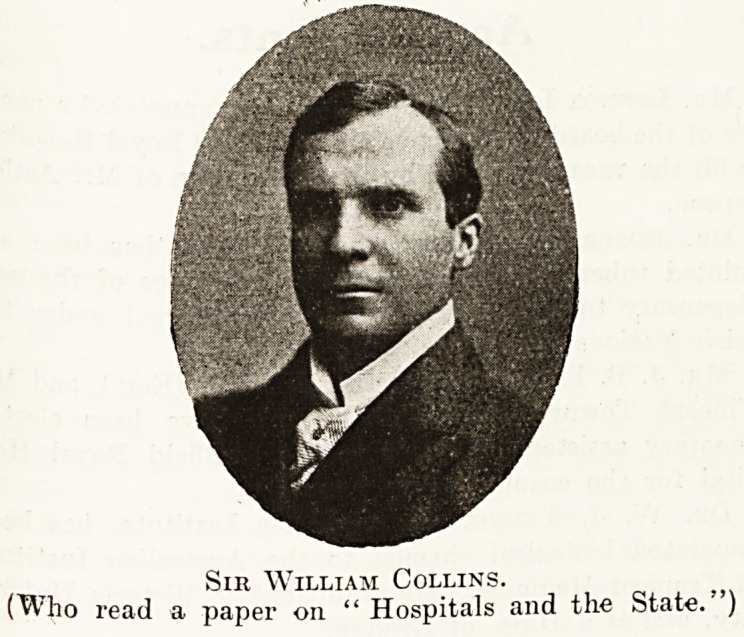


**Figure f2:**
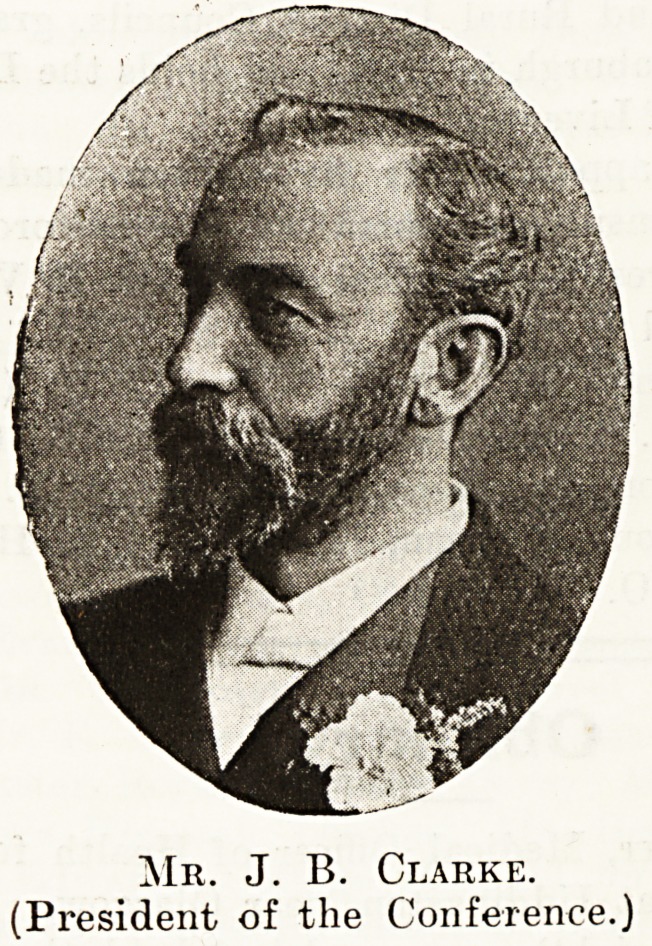


**Figure f3:**
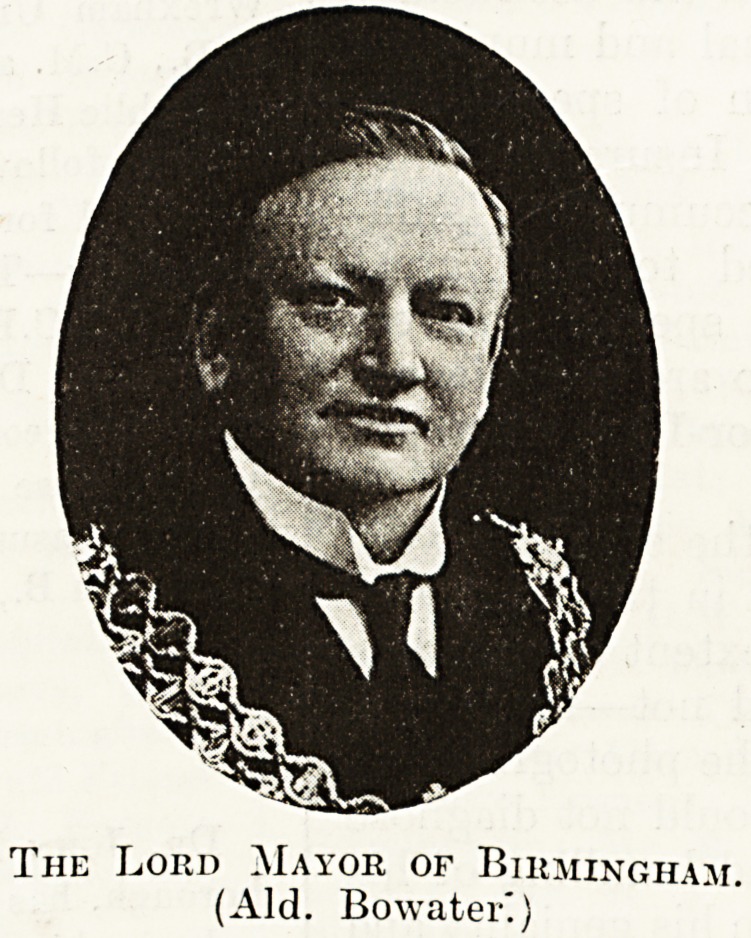


**Figure f4:**
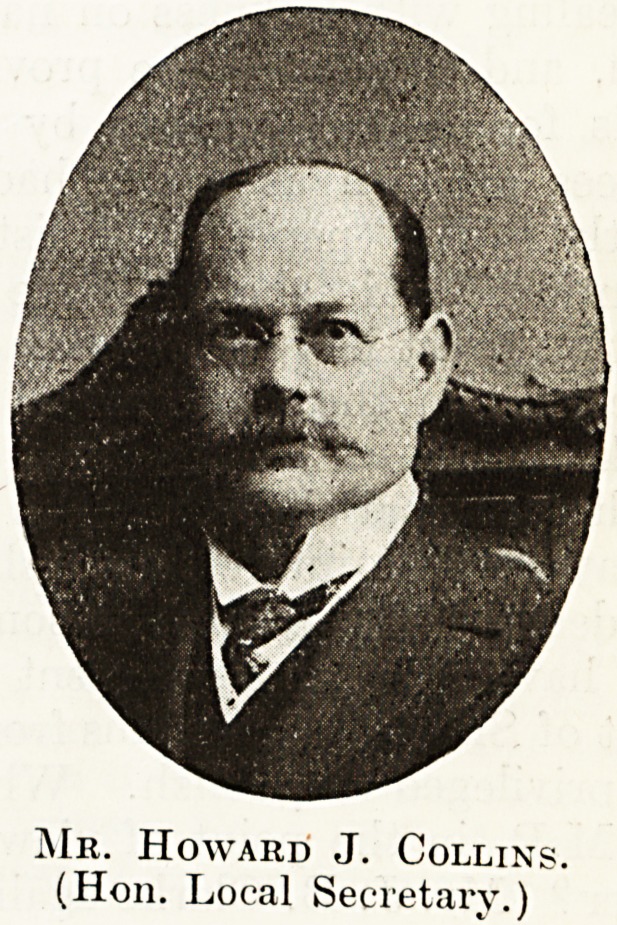


**Figure f5:**